# An exploration of suicidal ideation and attempts, and care use and unmet need among suicide-ideators in a Belgian population study

**DOI:** 10.1186/s12889-023-16630-7

**Published:** 2023-09-07

**Authors:** Eva Rens, Gwendolyn Portzky, Manuel Morrens, Geert Dom, Kris Van den Broeck, Mandy Gijzen

**Affiliations:** 1 Department of Family and Population Health (FAMPOP), University of Antwerp, 2610 Antwerp, Belgium; 2 Collaborative Antwerp Psychiatric Research Institute (CAPRI), University of Antwerp, 2610 Antwerp, Belgium; 3 Flemish Centre of Expertise in Suicide Prevention, Department of Head and Skin, Ghent University, 9000 Ghent, Belgium; 4 University Psychiatric Centre Duffel, 2570 Duffel, Belgium; 5 Multiversum Psychiatric Hospital, 2530 Boechout, Belgium

**Keywords:** Suicide, Suicidal ideation, Suicide attempts, Unmet mental health need, Help-seeking

## Abstract

**Background:**

Suicidal ideation, or thinking about death and suicide, is common across all layers of society. The aim of this paper is to add to the understanding of suicidal ideation in the general population, as well as help-seeking behaviors and perceived unmet mental health needs among those who report suicidal thoughts.

**Methods:**

The research is part of a representative population-based survey study of mental wellbeing in Antwerp (Flanders, Belgium) carried out in 2021. A total of 1202 participants between 15 and 80 years old answered the Ask Suicide-Screening Questions (ASQ), and an additional question about suicide plans. Participation was by invitation only and possible online or via a postal paper questionnaire. Univariate and multivariate logistic regression analyses were used to explore the association between both current suicidal ideation and self-reported lifetime suicide attempt with the sociodemographic factors age, gender, educational level, origin and financial distress. Moreover, formal care use for mental health was examined among those experiencing suicidal ideation, and logistic regression analyses were used to assess associated sociodemographic factors. Finally, perceived unmet mental health needs were assessed among suicide ideators.

**Results:**

The point-prevalence of suicidal ideation was 8.6% and was higher among younger age groups and individuals reporting financial distress. The lifetime-prevalence of suicide attempts is 6.5% and was higher in younger people and individuals with a primary educational level and with financial distress. About half (45.6%) of those with suicidal ideation consulted a professional for mental health problems in the past twelve months. Men and those with a primary educational level were less likely to seek help. Half of suicide ideators without care use perceived some need for mental health care, and a third of suicide ideators who used care perceived the obtained help as insufficient, resulting in a population prevalence of 3.6% suicide ideators with a fully or partially perceived unmet need.

**Conclusions:**

The prevalence of suicide attempts, suicidal ideation and unmet needs among suicide-ideators is high in this Belgian sample. Mental health care need perception in suicide ideators needs further investigation.

## Introduction

Suicide is a global public health issue and one of the leading causes of death, accounting for one in 100 deaths [1]. In 2019, the age-standardized suicide rate was 8.96 deaths per 100.000 people globally, and 10.5 deaths per 100.000 people in Europe [2]. Furthermore, it is estimated that there are more than 20 suicide attempts per suicide death [3]. Suicide is the result of a complex interaction of neurobiological and psychological susceptibilities, and it is often associated with stressful events and poor conditions such as loneliness [4, 5]. Suicidality is often considered a continuum, ranging from suicidal ideation to suicide attempts. ‘Suicidal ideation’ involves a range of thoughts, wishes and preoccupations with death and the possibility of ending one's own life [6]. Also within suicidal ideation, a distinction is often made between transient thoughts and passive ideation (i.e., thoughts about being death in general), and persistent ruminations and active ideation (i.e., concrete suicidal thoughts or plans) [7–9].

A meta-analysis found that the prevalence of passive suicidal ideation ranges from 2% point-prevalence to 11% for lifetime ideation in the general population, and even up to 47% for lifetime ideation in psychiatric samples [7]. This is in line with results from the World Mental Health Surveys carried out in 21 countries in the early 2000s, demonstrating that the lifetime prevalence of suicidal ideation, suicide plans and suicide attempts are 9%, 3% and 3%, respectively [10]. Between 2015 and 2019, a total 4.3% US adults reported suicidal thoughts, 1.3% a suicide plan and 0.6% a suicide attempt in the preceding year [11].

As can be expected, these are intercorrelated: among those with lifetime suicidal ideation, one in three also had a lifetime suicide plan and/or a suicide attempt [10]. Suicidal ideation is often transient, as some studies report that the majority of suicide ideators at baseline do no longer report suicidal thoughts after a follow-up period of one and a half year [12, 13]. However, suicidal ideation generally fluctuates greatly, even in a span of a few days, and stronger fluctuations were found to be associated with higher levels of suicidal ideation [14].

Although fewer than one in 200 ideators actually die from suicide, it remains a major risk factor for suicide [12]. People expressing suicidal ideation are four times more likely to die by suicide compared to non-suicide-ideators, with an absolute suicide risk of 0.2% in non-psychiatric and 1.4% in psychiatric populations, but the highest relative risks in non-psychiatric populations [15]. A past suicide attempt is an especially strong and robust risk factor for completed suicide, even up to thirty years after the first attempt [16]. A study using medical records found a prevalence of 5.4% suicide deaths in a cohort of 1490 individuals of whom a first suicide attempt was recorded during a 20 year period, of which 40.7% did not die on the first attempt [17].

It is commonly found that socio-demographic risk-factors of suicidal ideation and behavior include being female, younger age and lower socio-economic status [7, 8, 10, 18]. The relation between gender and suicide is complex. Suicidal ideation and nonfatal suicide attempts are higher in women, but men are two to four times more likely to die from suicide, often referred to as the ‘suicidal behavior gender paradox’ [19, 20]. One of the main reasons for this is that males use more lethal methods when committing suicide, but even when the same method is used, male suicide attempts are more often classified under ‘serious attempts’ where there is a clear intent to die [20, 21].

It is estimated that one in three people with suicidal thoughts (approximately one in two in high-income countries) seeks help for their mental health in a given year, although it is unclear what proportion of help seekers also explicitly seeks help for suicidality [22, 23]. This demonstrates the high unmet need among suicidal people, but formal help-seeking generally increases as suicide risk increases [24]. Key barriers to seeking help include a preference to solve problems on their own, a lack of perceived need for care, fear of stigmatization and stigmatizing attitudes. On the other hand, social support and mental health literacy facilitate professional help-seeking [23, 25].

A systematic review showed that 57% of all people who died from suicide had a lifetime mental health care contact, and 31% had a contact with mental health services in the final twelve months [26]. Male sex and both younger and older age are consistently associated with non-receipt of mental health services [27]. An analysis of US nationally representative data between 2008 and 2019 found that mental health care use among suicide attempters has not increased [28]. However, some people may also use other help sources for suicidality that fall outside of the traditional mental health field. For example, the internet nowadays offers numerous anonymous support resources which may have caused a ‘help seeking shift’, and there is some evidence that especially younger and higher-risk groups seek suicide-related help online [29].

Regarding perceived or subjective care needs, a study found that 35% of those who experienced suicidal ideation in the past twelve months perceived an unmet mental health need at some point. On the other hand, looking at those with suicidal ideation who did not receive any treatment during that period, two thirds did not perceive an unmet mental health need [30]. Another study found that differences in perceived unmet needs and health care use between persons with a common mental disorder with and without suicidal ideation were largely explained by severity of the symptomatology, as persons with suicidal ideation typically have more severe mental illness. Moreover, even when receiving mental health care, persons with suicidal ideation were more likely to perceive this care as insufficient or inadequate [31]. In 2019, a total of 46% of suicide attempters in the US reported needing mental health care but did not receive it, and this did not significantly change during the decade [28].

The study described in this paper examines the point prevalence and risk factors of suicidal ideation in a Belgian province (Antwerp) based on a population survey study, as well as the lifetime prevalence of self-reported suicide attempts, and twelve-month health care use for mental health and unmet need perception among suicide ideators. Suicide numbers are high in Belgium, with an age-standardized prevalence of 13.93 deaths per 100.000 people in 2019 [2]. Prior research from the four-yearly national Belgian health survey (2018) found that 13.9% of Belgians ever seriously thought about suicide that time, of whom 4.3% in a 12-month period. Four percent ever attempted suicide and two in thousand did so in the past twelve months [32].

However, it is unclear how many of Belgian suicide ideators had previous contacts with mental health services and perceive an unmet treatment need. The current paper explores the correlates of suicidal ideation and attempts, help seeking and the perception of unmet need among those with suicidal thoughts in Antwerp (Belgium), with the aim to add to the understanding of the (unmet) needs regarding suicide in the general population.

## Methods

As part of a research project which aims to assess the level of (unmet) mental health needs in the Antwerp Province in the Flemish region of Belgium, a sample of 5000 Antwerp citizens aged 15 to 80 years was invited to participate in a mental wellbeing survey between May and August 2021. The sample was randomly drawn from the national register and was stratified by gender, municipality, age and nationality (Belgian versus non-Belgian). Invited individuals were asked to fill in a self-report questionnaire and could participate online or through a paper questionnaire sent by postal mail. The paper questionnaire was available in the official language Dutch only, but the online questionnaire was available in six languages that are commonly spoken in Antwerp: Dutch, French, English, German, Polish and Arabic. Ethical approval was obtained from all participants.

The following socio-demographic information was included in the current study: age category (15 – 25y old, 26 – 39y old, 40 – 64y old, 65 – 80y old), gender (male, female), origin (geographic region of Europe, non-Europe), educational attainment (primary education i.e. no high school degree, secondary education or higher) and financial distress (self-reported financial difficulties or not).

Suicidal ideation was measured with five yes/no questions, of which four questions were adapted from the ultra-brief Ask Suicide Screening Questions (ASQ) Suicide Screening Toolkit from the US National Institute of Mental Health (NIMH), which is a validated tool for identifying adults and youth at elevated suicide risk in all clinical settings [33, 34]. Note that the question about ‘thoughts of killing yourself right now’ was not included as this is more of relevance in a clinical setting to assess suicide risk. Also, a question about suicide plans was added. The original ASQ uses the term ‘killing yourself’, but we decided to use ‘committing suicide’ in the English version as this is more in line with the common Dutch-language terminology that was used in the Dutch version. Current suicidal ideation was considered present when one or more of the four items about suicidal ideation in the past two weeks were answered positively. Question 5, lifetime suicide attempt, was not considered for current suicidal ideation. The items are mentioned in Table 1 in the Results section.

**Table 1:** Item scores on suicidal ideation and lifetime suicide attempt (*N* = 1202)

Item	%	N
Overall current suicidal ideation (item 1 – 4)	8.6%	103
* 1. In the past few weeks, did you wish you were dead?*	5.6%	68
* 2. In the past few weeks, have you felt that you or your family would be better off if you were dead?*	5.5%	67
* 3. In the past few weeks, have you had thoughts about committing suicide?*	5.3%	64
* 4. In the past few weeks, have you had plans about committing suicide?*	0.9%	11
Lifetime suicide attempt	6.5%	78

Health care use for mental health problems was assessed by asking the individuals whether they consulted a professional for their mental health in the past twelve months. Those reporting health care use for mental health were asked which professional was consulted (a general practitioner (GP), and/or a psychologist, and/or a psychiatrist) and whether they were prescribed medication for their mental health. Finally, questions about perceived unmet needs for mental health care were asked. Concretely, those with suicidal ideation who reported using health care for their mental health in the past twelve months were asked whether they thought the received help was sufficient. On the other hand, those who did not report using health care for mental health were asked whether they thought they needed help in the past twelve months but did not seek it. Those answering yes are defined as having an unmet need for mental health care.

The observations were weighed to match the Antwerp population using inverse probability weighting to correct for differences in response rate (i.e., larger weight for individuals from subgroups with higher non-response) between age categories, gender and nationality (Belgian versus non-Belgian) [35]. Descriptive characteristics of the sample and the prevalence of suicidal ideation, lifetime suicide attempts, health care use for mental health problems and perceived unmet mental health need are reported using weighted percentages.

Univariate and multivariate logistic regression analyses are used to assess the relationship between sociodemographic variables (sex, age, education, origin, financial distress) and the presence of suicidal ideation and lifetime suicide attempts in the general population. Next, logistic regression analyses were used to assess the sociodemographic associates of mental health care use among those reporting suicidal ideation. Crude odds ratios (COR) are presented for the univariate regression analyses, and adjusted odds ratios (AOR) for the multivariate analyses. Finally, Chi Square tests were used to examine the associations between perceived unmet needs and suicidal ideation.

## Results

A total of 1202 Antwerp citizens participated in the survey and had no missing demographic data or data about suicidality (24% of the invited sample). In the weighted sample, 49.7% of the participants were men, 10.8% has a non-European place of birth and 13.6% has no secondary education. The mean age is 45.5 years old.

The item scores are presented in Table 1. Overall, 103 persons or 8.6% of all participants experienced suicidal ideation in the past few weeks. A total of 44.2% of suicide ideators answered yes to only one suicidal ideation item, 18.6% to two items, 29.1% to three items and 8.1% to all four suicidal ideation items. Only 11 people (0.9%) reported suicidal plans in the past few weeks. A lifetime suicide attempt was reported by 78 individuals (6.5%). Among those reporting a suicide attempt, 63.1% has no current suicidal ideation, and 2.4% of the total sample reported both suicidal ideation and a lifetime suicide attempt.

The sociodemographic distribution of current suicidal ideation together with the results of the univariate and multivariate logistic regressions is shown in Table 2. No gender difference was found. Age is a strong risk factor, with a higher prevalence of suicidal ideation among younger age groups. In the multivariate model, the prevalence of suicidal ideation among people aged 65 and older (3.4%) is significantly lower than the prevalence among 26 to 44 year old’s (10.3%; OR = 3.60, 95% CI 1.58 – 8.19) and 15 to 25 year old’s (15.0%; OR = 5.68, 95% CI 2.38 – 13.54). Twelve percent of those without and eight percent of those with secondary education reported suicidal ideation, but this was not significant. Suicidal ideation did not differ between those with a European or non-European place of birth in the univariate analysis, but non-Europeans were significantly less likely to ideate suicide in the multivariate analysis (OR = 0.41, 95% CI 0.19—0.88). Finally, self-reported financial distress is a major risk factor of suicidal ideation: 6.6% of those without financial difficulties reported suicidal ideation, as compared to 18.4% of those with financial difficulties (OR = 4.15, 95% CI 2.59 – 6.64).

**Table 2:** The sociodemographic distribution and associated factors of suicidal ideation in the general population (*N* = 1202)

		n	%	COR	95% CI COR	AOR	95% CI AOR
Gender	Male (ref)	51/598	8.5				
	Female	53/604	8.8	1.03	.69 – 1.54	1.06	.70 – 1.61
Age in years***	65 – 80 (ref)	7/208	3.4				
	45 – 64	26/406	6.4	1.89	.82 – 4.35	1.80	.77 – 4.23
	26 – 44	40/387	10.3	3.14**	1.40 – 7.03	3.60**	1.58 – 8.19
	15 – 25	30/200	15.0	4.82***	2.10 – 11.06	5.68***	2.38 – 13.54
Education	Primary	20/162	12.3				
	Secondary or higher	82/1025	8.0	.63	.37 – 1.06	.97	.53 – 1.77
Origin*	Europe (ref)	94/1073	8.8				
	Non-Europe	10/130	7.7	.84	.42 – 1.68	.41*	.19—.88
Financial distress***	No (ref)	66/993	6.6				
	Yes	38/206	18.4	3.17***	2.06 – 4.89	4.15***	2.59 – 6.64

*COR* Crude odds ratio (univariate logistic regression), *AOR* Adjusted odds ratio (multivariate logistic regression), *p < .05, **p < .01, ***p < .001

The sociodemographic distribution of the presence of a lifetime suicide attempt and the results of the logistic regressions are shown in Table 3. There were no effects of gender or origin. People in the younger age groups were significantly more likely to report a lifetime suicide attempt. In the multivariate regression analysis, those between 26 and 44 years old had a higher odds of reporting a suicide attempt than the oldest age group (OR = 2.96, 95% CI 1.23 – 7.14). Moreover, people with a secondary education or higher are less likely to have attempted suicide in their life (OR = 0.38, 95% CI 0.21—0.70). The effect of financial distress is strongly significant with a three times higher odds of lifetime suicide attempts among those with self-reported financial problems (OR = 3.36, 95% CI 2.01 – 5.64).

**Table 3:** The sociodemographic distribution and associated factors of a lifetime suicide attempt in the general population (*N* = 1202)

		n	%	COR	95% CI OR	AOR	95% CI AOR
Gender	Male (ref)	32/598	5.4				
	Female	46/604	7.6	1.47	.92 – 2.34	1.61	.99 – 2.61
Age in years	65 – 80 (ref)	7/208	3.4				
	45 – 64	23/406	5.7	1.87	.78 – 4.47	1.98	.81 – 4.84
	26 – 44	31/387	8.0	2.61*	1.12 – 6.12	2.96*	1.23 – 7.14
	15 – 25	17/200	8.5	2.77*	1.11 – 6.92	2.28	.88 – 5.95
Education**	Primary (ref)	22/162	13.6				
	Secondary or higher	54/1025	5.3	.36***	.21 – .61	.38**	.21—.70
Origin	Europe (ref)	66/1073	6.2				
	Non-Europe	12/130	9.2	1.52	.80 – 2.92	.80	.40 – 1.62
Financial distress***	No (ref)	47/993	4.7				
	Yes	30/206	14.6	3.47***	2.14 – 5.64	3.36***	2.01 – 5.64

*COR* Crude odds ratio (univariate logistic regression), *AOR* Adjusted odds ratio (multivariate logistic regression), *p < .05, **p < .01, ***p < .001

More than one in six (17.1%, *n* = 206) of all participants used health care for their mental health in the past twelve months. A total of 22.8% (*n* = 47) of all people with a mental health related health care contact reported suicidal ideation. About half (*n* = 47, 45.6%) of those reporting suicidal ideation consulted a professional for mental health problems in the past twelve months, compared to 14.5% (*n* = 159) of those without suicidal ideation. Therefore, there are 4.7% people with suicidal ideation who do not use health care for their mental health in the total sample. Of all help seekers with suicidal ideation, 70.2% consulted a GP, 66.0% a psychologist, and 27.7% a psychiatrist. Moreover, 48.9% was also prescribed medication for their mental health.

Table 4 shows the sociodemographic distribution and associated factors of help-seeking among those reporting suicidal ideation. Note that the small subsample of suicide ideators (*N* = 103) raises very wide confidence intervals and the results should be interpreted taking into account this large statistical uncertainty. Sixty percent of women experiencing suicidal ideation sought help for their mental health, as compared to 28.2% of men (OR = 4.01, 95% CI 1.62 – 9.92). As regards educational level, 51.9% of people with at least a secondary degree as compared to 22.2% of those with a primary degree in the group of suicide ideators sought professional help (OR = 4.63, 95% CI 1.15 – 18.59).

**Table 4:** The sociodemographic distribution and associated factors of help-seeking in those reporting suicidal ideation (*N* = 103)

		n	%	COR	95% CI COR	AOR	95% CI AOR
Gender**	Male (ref)	11/39	28.2				
	Female	35/58	60.3	3.87**	1.62 – 9.28	4.01**	1.62 – 9.92
Age in years	65 – 80 (ref)	2/10	20.0				
	45 – 64	15/30	50.0	3.59	.51 – 24.96	2.18	.23 – 18.33
	26 – 44	17/31	54.8	4.80	.73 – 31.74	4.58	.57 – 36.49
	15 – 25	12/26	46.2	2.13	.31 – 14.70	2.56	.31 – 21.41
Education*	Primary (ref)	4/18	22.2				
	Secondary or higher	40/77	51.9	3.79*	1.14 – 12.53	4.63*	1.15 – 18.59
Origin	Europe (ref)	42/90	46.7				
	Non-Europe	4/7	57.1	1.52	.32 – 7.20	.36	.07 – 2.04
Financial distress	No (ref)	30/66	45.5				
	Yes	16/31	51.6	1.28	.54 – 3.01	1.40	.51 – 3.84

*COR* Crude odds ratio (univariate logistic regression), *AOR* Adjusted odds ratio (multivariate logistic regression), *p < .05, **p < .01, ***p < .001

Finally, questions were asked about perceived (unmet) needs for mental healthcare. Among those with suicidal ideation but no health care use for their mental health, half (50.0%, 28 out of 56) reported that they perceived some need for mental health care in the past twelve months but that they did not seek help. As a comparison, this prevalence is 9.9% (93 out of 940) among non-help-seekers without suicidal ideation, and a Chi square test showed that there is a significant association between suicidal ideation and reporting fully unmet mental health needs (X^2^ (1, *N* = 996) = 79.65, *p* < 0.001).

Secondly, those who consulted a professional in the past twelve months were asked whether they thought they were sufficiently helped with their mental health problem. Help-seekers with suicidal ideation were more likely to report that the help was insufficient (34.0%, 16 out of 47) compared to help-seekers without suicidal ideation (20.1%, 32 out of 159) ((X^2^ (1, *N* = 206) = 3.93, *p* = 0.047). As a total of 75 out of 103 suicide ideators either sought help or perceived a need for mental health care in the absence of help, it can be concluded that 72.8% of suicide ideators perceived some mental health need.

Overall, persons with suicidal ideation with a fully unmet need (no care use for mental health) make up 2.3% of the sample, and persons with suicidal ideation with a partially unmet need (insufficient care obtained) 1.3%, leading to a total population prevalence of 3.6% unmet suicidal ideation needs.

## Discussion

The study described in this paper explored the local prevalence and correlates of past-weeks suicidal ideation and lifetime suicide attempts in the general population in the province of Antwerp (Belgium), as well as help seeking for mental health and the perception of unmet mental health need among those experiencing suicidal ideation.

A strength of the study is the use of a representative probability sample. Moreover, participation was possible in six languages and both online and through paper-and-pencil, which allows vulnerable groups such as foreign-language immigrants and those without internet access to participate. However, some bias because of self-selection cannot be avoided, for example, because persons with mental health problems may be more likely to participate. Also, the smaller sample sizes in some subgroups raise large statistical uncertainty. The findings should only be interpreted in the context of descriptive and exploratory research.

Suicidal ideation is high in the sample, with a higher relative prevalence among younger people, people reporting financial difficulties and Europeans. Based on the item-scores, 5.3% and 0.9% respectively thought and planned to commit suicide in the past few weeks,. In comparison, 3.3% of the Flemish population (i.e., Flanders is the Belgian part where Antwerp is located) seriously thought about suicide in the past twelve months according to the national health survey in 2018, mostly women and young people [32]. A meta-analysis found that there is a point-prevalence of 2% of passive suicidal ideation in the general population worldwide [7]. It is unclear whether this is due to a general increase in suicidal ideation, given that many of the included prevalence studies are at least ten years old. The high level of suicidal ideation in women and younger age groups is in line with research reporting an alarming increasing trend in suicidal ideation in young and middle-aged women [36]. Another possible explanation is that suicidal ideation might be especially high in Belgium. As regards actual suicides, it is estimated that the Belgian suicide mortality rate is very high, about 1.7 times higher than the EU-average [37]. It must be mentioned that this should be interpreted with caution due to international differences in registration and data quality.

On the other hand, other studies also reported even higher level of suicidal ideation in 2021 and argue that this can at least partly be explained by the COVID-19. A systematic review reported a pooled prevalence of 11.5% of suicidal ideation in the general population during the early stages of COVID-19, but this includes twelve-month prevalence estimates as well [38]. Also in Belgium, several survey studies pointed to increased rates of suicidal ideation. In June 2021, which is during the study period, 10.5% of respondents reported having seriously considered suicide in the past 12 months, and in young people (18–29 years old) this percentage rises to 17% [39]. Regarding suicide attempts, 0.7% of all respondents and 2.0 of young people reported having attempted suicide in the past 12 months [39]. It should be noted that the use of non-probability samples during the COVID-19 pandemic may have influenced these findings, but nevertheless these numbers are high and suggest a small elevation in suicidality during the pandemic [40]. No official suicide mortality numbers during the COVID-19 pandemic in Belgium are available up to date, but registrations on suicides and attempts from the Federal Police showed no increase [41].

The prevalence of reporting ever having attempted suicide is very high in the sample. More than one in sixteen people (6.5%) claimed ever having attempted suicide, which is remarkably more than the number of the BHIS 2018, where 3.4% of Flemish people reported a lifetime suicide attempt. Also globally, 3% reported a suicide attempt in the World Mental Health Surveys twenty years ago [10]. The reasons for this discrepancy are unclear.

Among people reporting financial distress in our sample, even 14.6% ever attempted suicide. Given that a prior suicide attempt is considered the most critical risk factor for suicide in the general population, this group needs extra attention [17]. The same holds for lower educational level, which is another proxy of socio-economic status. Further research should examine if the high suicidality is explained by the financial stress itself, or (a combination of) other social vulnerability factors or possible associated risk factors such as lower social support or poor neighborhood.

As regards help seeking, it was found that half of those with current suicidal ideation reported using health services for their mental health in the past twelve months, especially a GP and/or psychologist, and half were also prescribed medication for a mental health problem. Persons with suicidal ideation make up more than one-fifth of the population of mental health care users. Men and people with a lower educational attainment who experience suicidal ideation are less likely to seek professional help.

The lower use of mental health services and higher level of unmet needs among men with mental health problems is a consistent finding in the literature [42–46]. In general, men are less likely to perceive a need for mental health care and have less favorable attitudes towards psychological openness and help-seeking for mental health problems [43]. This can be explained by self-stigma due to conformity to traditional masculine norms such as emotional control and toughness [46, 47]. In line with this, a study found that more men than women who died by suicide in the UK were not in contact with mental health services a year before dying [48].

It was found that 72.8% of suicide ideators perceived some need for mental health care. In comparison, in research on need perception among people with a common mental disorder, it was found that as much as two thirds of those who fulfill the criteria for a mental disorder did not perceive a mental health problem that needs care [49]. Moreover, half of suicide ideators who did not use any health care for their mental health did also not perceive a need for mental health treatment, and a third of help-seekers with suicidal thought the received help was insufficient. This leads to an estimated population prevalence of 3.6% perceived partially or fully unmet mental health needs among suicide ideators. As a reference, this number is 14% in the total sample (i.e., both with and without suicidal ideation), as one in ten people perceives a fully unmet mental health need and 4% perceives a partially unmet need [50]. Thus, those reporting suicidal ideation account for about one-quarter of all perceived unmet needs. Prior research in the same sample found that a preference to solve problems on their own is the most important barrier for not seeking any care, and cost of services is the most important barrier for not seeking additional care among people with a common mental disorder [50].

Low perceived need can reflect a lack of insight in one’s mental state, but on the other hand, some people with passive suicidal ideation can find a way to cope effectively with their symptoms, and spontaneous remission of suicidal thoughts and mental health problems is common [12, 51]. This implies that some people who do not perceive a need for mental health care are actually able to make an adequate estimation of their resilience or can sufficiently rely on their informal network. Nonetheless, it should be alarming when someone with more active suicidal ideation or who experiences significant disability due to mental illness or suicidality does not perceive a need for mental health care.

Another question is whether suicidality should, at least to some degree, be destigmatized so that persons who experience this feel more comfortable talking about it and seeking help. There is a reciprocal relationship between stigma and suicide: the negative perception that suicidal people are weak or selfish can cause self-stigma, which is a risk factor for suicide [52, 53]. Interestingly, a survey study compared attitudes from people in Flanders with those from people in the Netherlands, where suicide rates are lower, and found that that people from the Netherlands have more positive attitudes toward help seeking and experience less self-stigma and shame, which predicted formal as well as informal help seeking intentions [54]. Also suicide literacy was found to be significantly associated with more positive help-seeking attitudes, which also demonstrates the need for the promotion of suicide literacy [55].

On the other hand, some studies illustrate that suicide normalization or an overly liberal view of suicidality can be dangerous as well [53]. For example, adolescents who believed suicidal ideation and attempts to be more widespread among peers (i.e., elevated suicide ‘descriptive norms’) were also more likely to endorse suicidal ideation and attempts [56]. Given that lower suicide stigma is associated with higher suicide normalization, interventions should improve attitudes towards persons affected by suicidality that avoid conveying a message that suicide is an acceptable solution [53].

Although the study is not novel in design and several risk factors for suicidal ideation are already known, the study is relevant to the local insight into suicidal ideation. However, studies about care need perception among suicide ideators are rather scarce. More research is needed on the perception of (unmet) needs for mental health services among persons with suicidal ideation who do not seek help, and on the satisfaction and perceived sufficiency of the help among help-seekers, and what factors underlie these partially and fully unmet mental health needs among people experiencing suicidal ideation. Suicidal ideation has been addressed here in binary terms, but further research should also examine whether there are substantial differences between different levels of suicidal ideation.

## Conclusions

This survey study examined suicidal ideation, health care use for mental health and perceived unmet needs for mental health care in a representative general population sample in Antwerp, Belgium. Suicidal ideation was measured with four items which were a combination of past-weeks passive (e.g., dead wish) and active (e.g., thinking about actually committing suicide) ideation, and a question about lifetime suicide attempt was included. Key findings include that 8.6% experienced current suicidal ideation, and 6.5% ever attempted suicide. Suicidal ideation was higher among younger people and those with financial problems, and no gender effect was found. People aged 65 or older and people with a higher educational attainment and no financial distress were less likely to have ever attempted suicide. The demographic profiles of suicide attempters and ideators are generally similar in the sample. Half of suicide ideators did not consult a professional for their mental health, and half of those did not perceive a need for this as well. Among those who did consult a professional, a third perceived this help as insufficient. The perceived fully unmet and partially unmet needs for suicidal ideation in the general population are 2.3% and 1.3%, respectively.

It can be concluded that suicidal ideation is a serious and widespread public mental health problem in all age categories. Future research needs to further investigate suicidal ideation in the general public, and especially on how passive and active ideation and plans, attempts and completed suicides are related. Also, much remains unclear about (unmet) mental health treatment need perception among people with different levels of suicidality. Making suicidality discussable and eliminating barriers in seeking both formal and informal support are important, particularly among men and socially vulnerable groups.

## Data Availability

The datasets generated and analysed during the current study are not publicly available due to privacy constraints but are available from the corresponding author on reasonable request.
